# What matters to cardiac patients? The impact of linking life goals to health goals on patients' intention‐to‐change‐lifestyle: an online experiment

**DOI:** 10.1111/bjhp.70056

**Published:** 2026-01-30

**Authors:** Renée V. H. IJzerman, Rosalie van der Vaart, Linda D. Breeman, Inge van den Broek, Elise Dusseldorp, Roderik Kraaijenhagen, Thomas Reijnders, Andrea W. M. Evers, Wilma J. M. Scholte op Reimer, Veronica R. Janssen, the BENEFIT Consortium

**Affiliations:** ^1^ Health, Medical and Neuropsychology Unit Leiden University Leiden The Netherlands; ^2^ Department of Cardiology Amsterdam UMC, University of Amsterdam Amsterdam The Netherlands; ^3^ Centre of Expertise Health Innovation The Hague University of Applied Sciences Den Haag The Netherlands; ^4^ Harteraad Den Haag The Netherlands; ^5^ Methodology and Statistics, Institute of Psychology Leiden University Leiden The Netherlands; ^6^ NDDO Institute for Prevention and Early Diagnostics (NIPED) Amsterdam The Netherlands; ^7^ Vital10 Amsterdam The Netherlands; ^8^ Medical Delta (Partnership between Leiden University, Delft University of Technology, and Erasmus University) Delft The Netherlands; ^9^ Research Group Chronic Diseases HU University of Applied Sciences Utrecht The Netherlands; ^10^ Department of Cardiology Leiden University Medical Center Leiden The Netherlands

**Keywords:** behaviour change, cardiac rehabilitation, goal setting, intention, intention‐to‐change‐lifestyle, lifestyle change, secondary prevention

## Abstract

**Objectives:**

Guidelines advocate goal setting for promoting lifestyle changes in cardiovascular disease (CVD) patients. This study investigates 1) preferences in health and life goal domains in CVD patients, 2) the impact of linking life goals to health goals on intention‐to‐change‐lifestyle and explores 3) socio‐demographic and health‐related variables influencing intention‐to‐change‐lifestyle.

**Design:**

Online experimental study.

**Methods:**

Patients (*N* = 629, mean age 66.6; 39% female) were randomized to *health‐goal‐group* (*HG*) or *life‐and‐health‐goal‐group* (*LHG*). *HG* set a health goal, and *LHG* first established a life goal and then set a supporting health goal. Directly after goal setting, the primary outcome, intention‐to‐change‐lifestyle, was measured and analysed using logistic regression (high: 9–10 vs. lower: ≤8.5), as were the secondary outcomes.

**Results:**

Exercise goals were most frequently selected in *LHG* (66.0%) *and HG* (66.9%). Preference for selecting stress management was significantly higher in *LHG* (17.3%) than *HG* (9.3%), *χ* ^2^(1) = 8.85, *p* = .003; OR = 2.05, 95%CI [1.27–3.30]. The direct effect of goal‐setting condition on intention‐to‐change‐lifestyle was non‐significant (OR = .98, 95%CI [.71–1.34], *p* = .88). In exploratory analyses, lower‐ and medium‐educated patients showed significantly higher intention when life and health goals were linked (OR = 2.55, 95%CI [1.03–6.27], *p* = .04, and OR = 2.47, 95%CI [1.15–5.30], *p* = .02, respectively). Perceived meaning in life was positively associated with intention.

**Conclusions:**

No main effect of goal‐setting condition on intention‐to‐change‐lifestyle was found. Linking life goals to health goals increased preference for stress management and, in exploratory analyses, was associated with higher intention‐to‐change‐lifestyle among lower‐ and medium‐educated patients. Findings emphasize the relevance of personalized, value‐based goal setting within cardiac rehabilitation.

**Statement of Contribution**

**What is already known on this subject?**

- Goal setting is widely used in cardiac rehabilitation to promote lifestyle change.
- Linking life goals to health goals may enhance motivation and adherence to behaviour change.
- Lower socioeconomic position is associated with lower engagement in health behaviour change.

**What does this study add?**

- Patients linking life to health goals show a greater preference for addressing stress management.
- While no main effect of goal‐setting condition on intention‐to‐change‐lifestyle was found, low and medium‐educated patients had significantly higher intention when linking life and health goals.
- Personalized, value‐based goal setting is crucial for effective cardiac rehabilitation treatment.

## INTRODUCTION

Cardiovascular disease (CVD) remains the leading cause of global morbidity and mortality, underscoring the critical need for effective management strategies to enhance secondary prevention (Kotseva et al., 2019; Visseren et al., 2021). Within cardiac rehabilitation (CR), health goal setting has shown improved disease management outcomes (Conn et al., 2009). Recognizing this, American and European guidelines on preventing cardiovascular disease in clinical practice advocate for health goal setting to realize behaviour change in CVD patients (Arnett et al., 2019; Visseren et al., 2021). Building on these guidelines, a recent American Heart Association publication reaffirmed the significance of person‐centred care in clinical practice, advocating for various models prioritizing this approach. Central to the person‐centred care approach is the collaborative establishment of health goals by clinicians and patients, augmented by integrating the patient's narrative into the assessment, jointly creating health care plans and continuously reassessing and adjusting patient goals to align with the patient's evolving needs and preferences (Rossi et al., 2023).

While health goal setting is widely recommended, emerging evidence suggests that integrating health goals with life goals could enhance effectiveness in CR (Janssen et al., 2014; Wade, 1999). Life goals, which are broad objectives that individuals strive to accomplish, maintain or avoid (e.g., ‘Spending more time with family’, ‘Living independently’) (Sivaraman Nair, 2003), reflect what matters most to people; they are indicative of a person's meaning in life. Meaning in life can be described as subjective judgements on whether life is ‘worthwhile and significant, comprehensible and make sense and marked by the embrace or pursuit of one or more highly valued, overarching purposes or missions’ (p. 961) (Snyder et al., 2016). Health goals, by contrast, tend to be more specific targets or behaviours (e.g., ‘Walking for 30 minutes a day’ (so I can stay active enough to go on family outings), or ‘Doing a daily set of balance exercises’ (so I can continue living independently)), chosen to support these broader life goals.

According to goal‐setting theories, goals are hierarchically organized (Höchli et al., 2018). *Superordinate life goals* are abstract and long‐term, *intermediate goals* represent broad behavioural approaches and *subordinate health goals* are concrete, short‐term actions (Höchli et al., 2018; Janssen et al., 2014). These different levels influence each other dynamically, reinforcing top‐down (meaning‐driven) and bottom‐up (behaviour‐driven) mechanisms (Kruglanski et al., 2002; Shah & Kruglanski, 2003). Research suggests that linking life goals to health goals may enhance motivation and dedication to pursue the defined goals (Höchli et al., 2018; Klein et al., 1999), allowing individuals to benefit from the combination of various goal levels. Additionally, aligning health goals to life goals has shown potential in increasing *restorative* health behaviour (e.g., exercise regularly) and decreasing *deteriorative* health behaviour (e.g., smoking) (Kim et al., 2019; Kubzansky et al., 2018). However, most studies predominantly involve complex interventions, making it challenging to isolate the direct effect of linking life goals to health goals, especially for CVD patients. Furthermore, to our knowledge, little is known about the specific life and health goals that CVD patients prioritize, which is crucial for designing patient‐centred interventions.

The study has several objectives. First, we explore CVD patients' preferences in selecting subordinate health goal domains and superordinate life goal domains. Second, we investigate the impact of linking life goals to health goals on intention‐to‐change‐lifestyle, given that behavioural intention is commonly regarded as a predictor of actual behaviour (Sheeran, 2002). Intention can be understood as the subjective probability that one will engage in specific behaviour, reflecting the strength of one's commitment to act or one's motivational readiness or commitment to adopt new health behaviours (Fishbein & Ajzen, 2010). In the present study, intention‐to‐change‐lifestyle is operationalized as our primary outcome measure, reflecting the degree to which patients are committed to adopt or maintain healthy lifestyle behaviours. We hypothesize that linking life goals to health goals has the most favourable effect on patients' intention to change their lifestyle, compared to setting a health goal alone. Third, we conduct exploratory analyses to examine whether known socio‐demographic and health‐related factors, such as education level (Shi et al., 2023), and perceived meaning in life (Vos, 2021), are associated with intention‐to‐change‐lifestyle in our study population, and whether they modify the effect of goal‐setting condition on intention‐to‐change‐lifestyle.

## MATERIALS AND METHODS

### Study design

**FIGURE 1:**
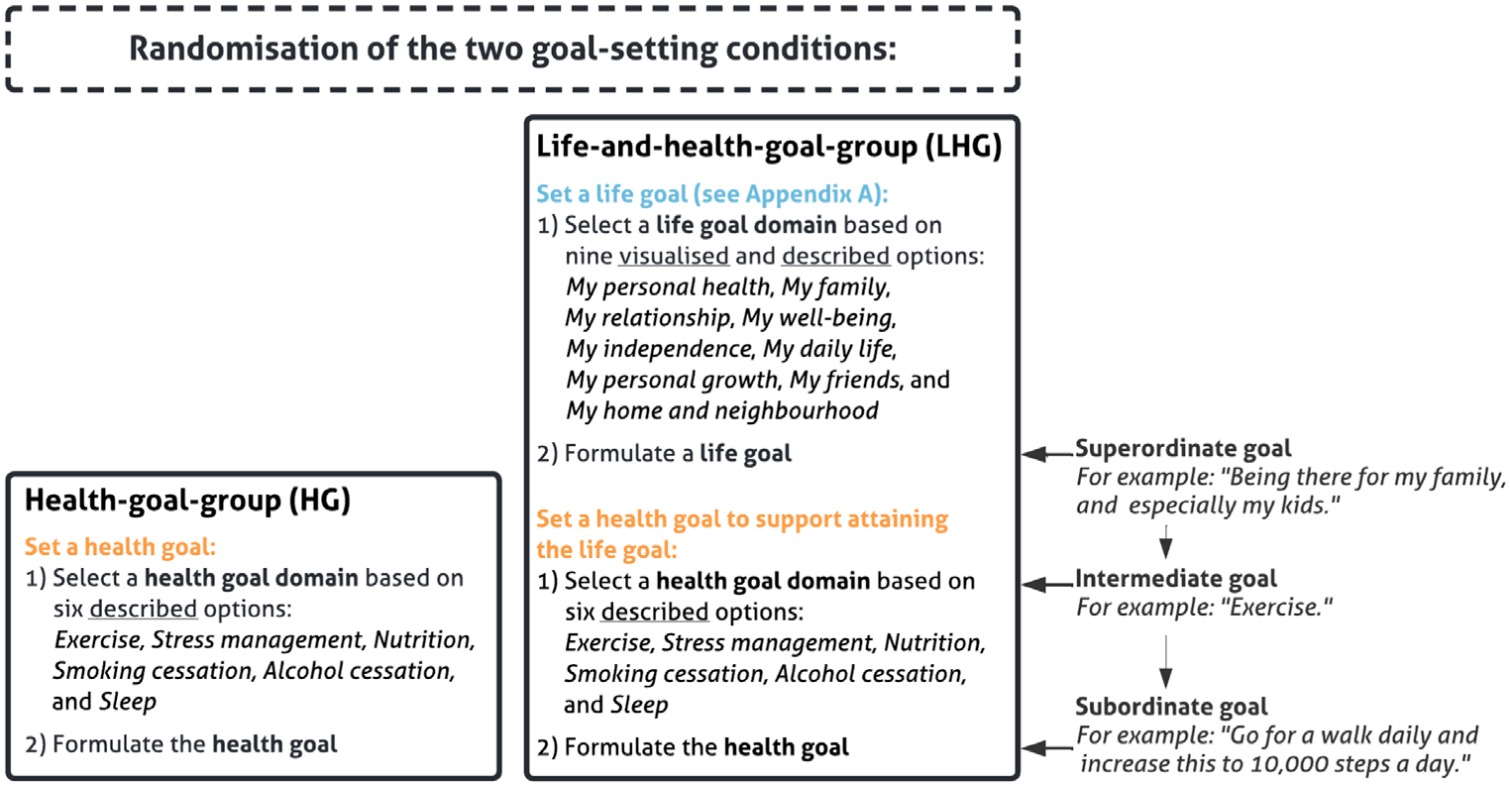
Patient randomization.

We conducted a randomized online experimental study to assess the effectiveness of linking superordinate life goals to subordinate health goals on CVD patients' intention‐to‐change‐lifestyle behaviour. Patients were randomly assigned (1:1 randomization) to one of the two goal‐setting conditions: the health‐goal‐group (HG, *n* = 323) or the life‐and‐health‐goal‐group (LHG, *n* = 306) (see Figure 1). Randomization was conducted within the online survey platform (Qualtrics) using an automated random allocation function. In the HG group, patients set a subordinate health goal (hereafter referred to as health goal), within a specific lifestyle domain (e.g., exercise, nutrition, stress management), while those in the LHG group set a superordinate life goal (hereafter referred to as life goal) followed by a health goal within a specific lifestyle domain to support attaining their life goal. The life goal setting was a structured reflection and had a visualization of life goals before setting a health goal. Directly afterwards, the primary outcome intention‐to‐change‐lifestyle was assessed.

### Study participants and procedure

Patients were members of the patient panel of Harteraad (the Dutch patient association for CVD), a voluntary database of approximately 2600 CVD patients. Recruitment was facilitated through an advertisement on the website of Harteraad, posted during the first two weeks of April 2021. The advertisement explained the study's purpose, duration, participation procedure, data protection and included the survey link. Before participation, all individuals received an information letter specifying that only people with a current or previous heart and/or vascular condition were eligible. Diagnoses were self‐reported. Participation in the study occurred at once, without a break. Informed consent was obtained at the start of the survey.

Inclusion criteria were being a Dutch‐speaking adult (≥ 18 years old) with a CVD diagnosis. Individuals were excluded if they were younger than 18 years of age, did not have a confirmed CVD diagnosis, had insufficient proficiency in Dutch to understand study materials or were unwilling or unable to provide informed consent. Eligibility and diagnosis were self‐reported; medical record verification was not possible in this online study.

A priori power analysis (using G*Power) indicated a minimum of 197 patients (approximately 99 per condition) to detect a significant difference in intention‐to‐change‐lifestyle (two‐tailed, α = .05, *power* = .80).

The online experimental survey (Qualtrics) consisted of three parts. First, all patients provided demographic and health‐related information (age, gender, education level, self‐reported CVD diagnosis). Second, in the randomized goal‐setting task (Figure 1), patients were assigned to either the HG group or LHG group. This ensured that assignment was unbiased and not influenced by individual preferences or prior experiences (e.g., previous participation in a CR programme). To support patients in determining their health goal, both groups were shown three illustrative examples within their chosen lifestyle domain (entry‐level, advanced and maintenance).

The LHG formulated their life goal by answering the first three questions from the Dutch shared decision‐making tool *Wat ertoe doet* (Harteraad, 2018) (meaning: *What matters*) (Appendix S1), which involved guided reflection and visual selection of life domains. The tool comprised different sequential screens: (1) patients viewed image tiles of nine life domains (e.g., family, personal health, independence, well‐being) and selected up to three images representing what is important in their life; (2) from these, they selected one image that is important to them now; (3) they then typed, in a free‐text field, what they want to be able to do within that domain (example prompts disappeared once typing started). This structured tool was designed to help patients clarify their overarching life goals before setting a health goal. The tool was systematically developed and tested extensively with different patient groups (Vroonland et al., 2019) and aims to support shared decision‐making by helping patients clarify what matters to them in life and how to link their life goals to their health goals.

In contrast, the HG group set a health goal without using the *Wat ertoe doet* tool first. This way, the HG group was used as an active usual‐care comparator.

In the third and final part of the survey, all patients completed the primary outcome variable intention‐to‐change‐lifestyle (two items) and the variable meaning in life (six items). After completing the survey, patients were debriefed about the study design.

### Measures

#### Primary outcome

Guided by the Reasoned Action Approach (Fishbein & Ajzen, 2010), the primary outcome variable intention‐to‐change‐lifestyle was assessed using two items to capture different aspects of intention related to health behaviour change. The first item, ‘I want to start carrying out my health goal or continue maintaining my health goal’, reflects the individual's attitude towards the behaviour. This item reflects the personal motivation or desire to engage in or maintain a health‐related behaviour, rooted in the belief that the action will lead to a positive outcome. The second item, ‘I intend to start carrying out my health goal or continue maintaining my health goal’, is more closely aligned with actual intention to perform the behaviour, which reflects a commitment to act. Using both items therefore allows for a nuanced understanding of the patients’ intention‐to‐change‐lifestyle. Both statements were rated via a 0–10 visual analogue scale (VAS) (Funke & Reips, 2012), representing a continuum between completely disagree (0) and completely agree (10). The scores on these two items were averaged to obtain a score on the scale ‘intention‐to‐change‐lifestyle’ (Cronbach's alpha = .83, inter‐item correlation = .71). Due to a very negatively skewed distribution (Appendix S2), and transformations to normal were not effective, intention was analysed dichotomously using a median split: scores up to 8.5 were considered lower intention, whereas scores from 9 up to 10 indicated high intention. This allowed for a clearer interpretation of effects.

#### Secondary outcomes

Socio‐demographic (age, gender, education) and health‐related characteristics (self‐reported cardiovascular diagnosis) were measured once at baseline (pre‐randomization). Meaning in life was collected once at the end of the survey (post‐randomization) as a potential moderator.

Gender identification was answered via three multiple‐choice options: male, female or other. Age was entered in years by using numerical input and subsequently categorized for descriptive purposes only into four groups: 18–40 years, 41–60 years, 61–80 years, 81 and older. Education level was assessed based on nine multiple‐choice options, resulting in three education‐level categories: low (no education, primary education, pre‐vocational secondary education and other), medium (vocational education, senior general secondary education and pre‐university education) and high (higher vocational education and university), based on the Dutch Standard Education Classification 2021 Edition 2022/’23 (Centraal Bureau voor de Statistiek, 2022). In consultation with an experience expert from Harteraad, patients were asked to self‐report their cardiovascular diagnosis through three accessible multiple‐choice options: cardiac disease, vascular disease or cardiovascular disease. When unknown, one was able to leave the question unanswered. Self‐reported diagnoses have been widely used in epidemiological research when medical records are unavailable. While this method may introduce some uncertainty, it provides valuable insight into patient‐perceived diagnoses. Finally, meaning in life was measured via a Dutch translation of the Meaning in Life Questionnaire‐Short Form (MLQ‐SF) (Steger et al., 2006; Steger & Samman, 2012). The questionnaire has two subscales Search and Presence, each containing three statements which were rated via a 4‐point Likert scale, ranged not at all true to completely true.

### Statistical analysis

IBM SPSS Statistics V.25 was used for statistical analyses. To address our first objective, assessing preferences in health goals and life goals, we explored preferred life goal domains among LHG patients and health goal domains among both HG and LHG patients and performed chi‐square tests to examine the association between goal‐setting condition and selected health goal domains, identifying significant differences. All formulated health goals were categorized strictly based on the observed behaviour, regardless of the stated intention or selected domain. In cases where multiple or cross‐domain goals were provided, only the first stated behaviour was used and assigned to the corresponding category. This approach ensured consistency and avoided reclassification based on inferred intention.

For the second objective, assessing the effect of goal‐setting condition on intention‐to‐change‐lifestyle, logistic regression analysis was performed to examine the effect of the goal‐setting group (LHG vs. HG) on the probability of expressing ‘high intention’ to change lifestyle. Subsequently, as part of our third objective, conducting an exploratory investigation into socio‐demographic and health‐related factors that may influence intention‐to‐change‐lifestyle, we examined whether age, gender (reference group: man), education level (reference group: high education level), cardiovascular diagnosis (reference group: cardiovascular disease) and meaning in life were modifiers of the effect of goal‐setting group on intention, by adding its simple effect (as block 2) and the interaction effect with goal‐setting condition (as block 3) to the logistic regression model. In Table 3 of the results section, the results from each block will be displayed together (block 1: goal‐setting condition, block 2: main effects of potential modifiers, block 3: interaction effects with goal‐setting condition) to maintain focus on the key effects from each stage of the analysis. A *p*‐value of .05 or lower was considered statistically significant.

### Data management and ethical procedures

All data were collected electronically through web‐based data entry using Qualtrics and stored under the provisions of the National Data Protection Regulations. This study was approved by the Psychology Research Ethics Committee of Leiden University (reference number: 2021‐03‐10‐A.W.M. Evers‐V1‐3067). Digital informed consent was obtained from each patient prior to participation; the survey's first page explained the survey's content and concluded with informed consent.

## RESULTS

### Patient characteristics

Of the 750 patients who started the survey after giving informed consent, 629 (84%) completed the survey. Among these, HG had *n* = 323 (51.4%) and LHG had *n* = 306 (48.6%). This 17‐participant difference resulted from dropout at different stages of the survey. Detailed patient characteristics are shown in Table 1. Most patients identified as male (60.4%) with an average age of 66.6 years (SD: 10.2). Patients’ education levels were categorized as low (19.7%), medium (23.8%) and high (47.5%). The overall perceived meaning in life (range 1–4) was rated at 2.67 (SD: .55). The subscale ‘search for meaning in life’ scored 2.42 (.76), while the subscale ‘presence of meaning in life’ scored slightly higher at 2.92 (.68). At the single, post‐task assessment, primary outcome intention‐to‐change‐lifestyle (range 0–10) was high on average (M = 8.4 on a 0–10 scale, SD: 1.6), with 47.1% scoring 9–10.

**TABLE 1:** Patient characteristics.

Characteristics	Total	HG	LHG	Statistical comparison^d^	*p*‐value
*N*	629	323	306		
Age (years (SD))	66.6 (10.2)	66.9 (10.2)	66.3 (10.2)	*t*(627) = .70	.485
18–40 years	9 (1.4)	6 (1.9)	3 (1.0)		
41–60 years	142 (22.6)	68 (21.0)	74 (24.2)		
61–80 years	440 (70.0)	229 (70.9)	211 (68.9)		
81 and older	38 (6.0)	20 (6.2)	18 (5.9)		
Gender				*χ* ^*2*^(1) = .71	.399
Male	380 (60.4)	200 (61.9)	180 (58.8)		
Female	248 (39.4)	122 (37.8)	126 (41.2)		
Other	1 (.2)	1 (.3)	0 (0)		
Education level				*χ* ^*2*^(2) = 2.4	.302
Low	124 (19.7)	71 (22.0)	53 (17.3)		
Medium	206 (32.8)	100 (30.9)	106 (34.6)		
High	298 (47.5)	152 (47.1)	146 (47.7)		
Cardiovascular diagnosis^a^				*χ* ^*2*^(2) = 1.75	.417
Cardiac disease	304 (48.3)	160 (49.5)	144 (47.1)		
Vascular disease	118 (18.8)	54 (16.7)	64 (20.9)		
Cardiovascular disease	189 (30.0)	99 (30.7)	90 (29.4)		
No response	18 (2.9)	10 (3.1)	8 (2.6)		
Presence of meaning in life	2.9 (.7)	2.9 (.7)	3.0 (.7)		
Perceived meaning in life^b^	2.7 (.6)	2.7 (.6)	2.7 (.5)	*t*(627) = .73	.468
Search for meaning in life	2.4 (.8)	2.5 (.8)	2.3 (.7)		
Intention overall score^c^	8.4 (1.6)	8.4 (1.6)	8.4 (1.6)	*t*(627) = −.36	.717
Lower intention (scores 0 up to 8.5)	333 (52.9)	172 (53.3)	161 (52.6)		
High intention (scores 9 up to 10)	296 (47.1)	151 (46.7)	145 (47.4)		

*Note*: Data are presented as the number in proportion *n* (%) or mean (SD), unless stated otherwise.

Abbreviations: HG, health‐goal‐group; intention, intention‐to‐change‐lifestyle; LHG, life‐and‐health‐goal‐group.

^a^ Self‐reported cardiovascular diagnosis; if unknown, no response was recorded.

^b^ Assessed using a 4‐point Likert scale (not at all true [1] to completely true [4]) (Steger et al., 2006; Steger & Samman, 2012).

^c^ Assessed using 0–10 visual analogue scale ((VAS), ranging from completely disagree [0] and completely agree [10]) (Funke & Reips, 2012).

^d^ Group differences (HG vs. LHG) were tested using independent‐samples *t*‐tests for continuous variables and chi‐square tests for categorical variables, as reported in the ‘Statistical comparison’ column.

### Objective 1: Life goal and health goal domain preferences

#### Life goals

Patients in LHG most often preferred the domain *My personal health* (*n* = 106) as their life goal domain, such as ‘Being able to move well’ and ‘Being able to continue doing what I have been doing’. The second most popular domain was *My family* (*n* = 97), for example, ‘Being and remaining an active mother and grandmother’ and ‘Seeing my grandchildren grow up’. The third most popular domain was *My relationship* (*n* = 36), such as ‘Paying enough attention to each other’ and ‘Growing old together’. Appendix S3 displays life goal domains and example formulations.

#### Health goals

Chi‐square test showed a significant association between goal‐setting condition and health goal domain (Table 2), *χ* ^2^(5) = 12.40, *p* = .03. In both LHG and HG, *Exercise* was the most frequently selected health goal domain (LHG: 66.0%, HG: 66.9%). Patients set exercise goals such as ‘Doing something in the garden every day. Large garden!’ or ‘One hour of daily exercise (walking, cycling, exercises)’. Additionally, the prevalence of selecting *Stress management* was higher in LHG (17.3%; *n* = 53) than in HG (9.3%; *n* = 30), *χ* ^2^(1) = 8.85, *p* = .003; OR = 2.05; 95% CI [1.27–3.30]. Stress management goals were, for example, ‘No phone or anything, just doing my thing without being bothered’ and ‘Enjoying nature, art and music’. Appendix S4 displays health goal domain prevalence and example formulations. Appendix S5 visualizes the distribution of the selected life goal domains and health goal domains across both goal‐setting conditions.

**TABLE 2:** Health goal domain preferences, divided by goal‐setting condition.

Health goal domain	LHG (*n* = 306), *n* (%)	HG (*n* = 323), *n* (%)	*χ* ^*2*^	Effect size (Cramer's *V*)	OR (LHG vs. HG); 95% CI
Exercise	202 (66.0)	216 (66.9)	.05	.01	.96; [.69–1.34]
Stress management	53 (17.3)	30 (9.3)	8.85*	.12	2.05; [1.27–3.30]
Nutrition	38 (12.4)	53 (16.4)	2.02	.06	.72; [.46–1.13]
Sleep	6 (2.0)	9 (2.8)	.46	.03	.70; [.25–1.98]
Alcohol cessation	4 (1.3)	8 (2.5)	1.15	.04	.52; [.16–1.75]
Smoking cessation	3 (1.0)	7 (2.2)	1.42	.05	.45; [.12–1.74]

*Note*: The *χ* ^2^ (2 × 6) test assesses the overall association between the condition (LHG vs. HG) and domain. Row‐wise *χ* ^2^(1) and OR (LHG vs. HG) compare, per domain, how often it was selected in LHG versus HG. 95% confidence intervals (CI) are shown; Cramer's *V* indicates effect size.

* *p* = .003.

### Objective 2: Effect of goal‐setting group on intention‐to‐change‐lifestyle

Logistic regression analysis (block 1) showed no significant main effect of goal‐setting condition (LHG vs. HG) on intention‐to‐change‐lifestyle, OR = .98; 95% CI [.71–1.34], *p* = .88.

### Objective 3: Effect of socio‐demographic and health‐related variables on intention‐to‐change‐lifestyle

**FIGURE 2:**
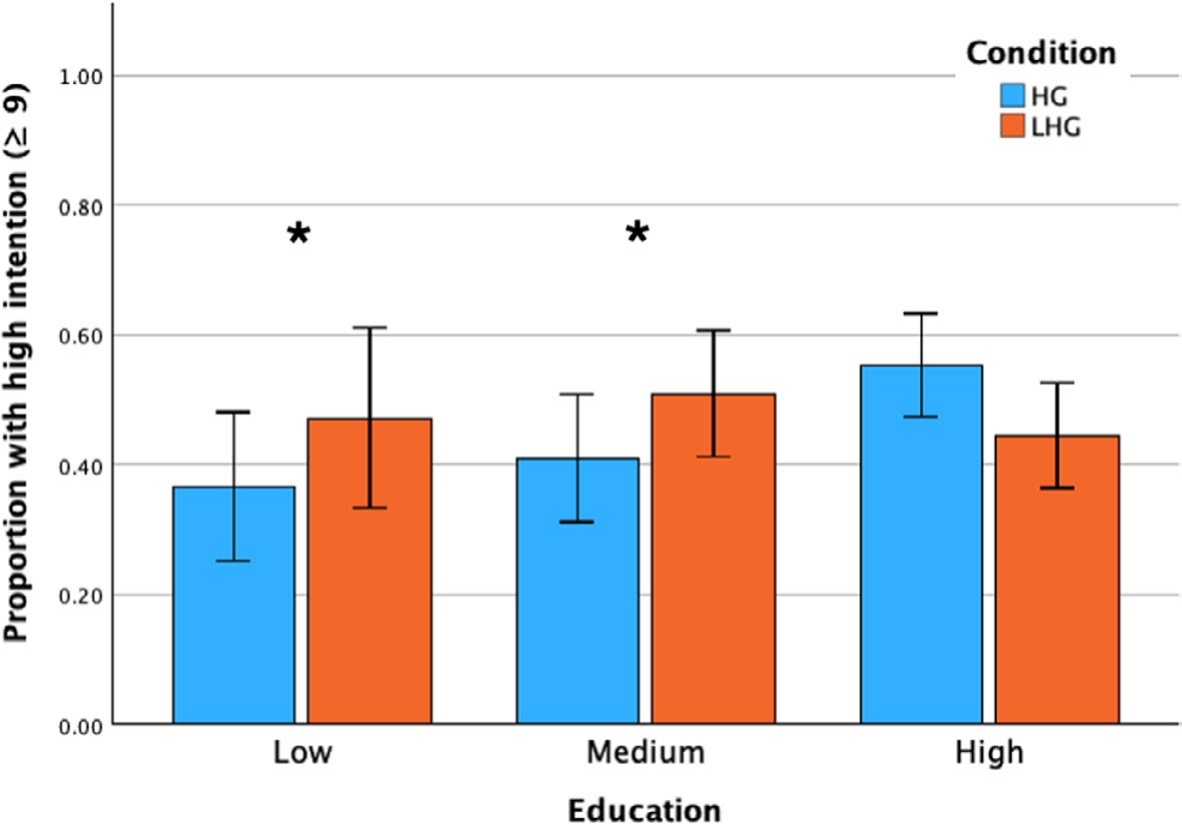
Proportion of patients with high intention (scores 9–10) by education and goal‐setting condition. HG = health‐goal group; LHG = life‐and‐health‐goal group. Error bars show 95% confidence intervals. Asterisks indicate significant differences between HG and LHG within education level (*p* < .05).

Logistic regression analysis (block 2) showed that lower‐educated patients were less likely to express higher intention‐to‐change‐lifestyle compared to higher‐educated patients (OR = .59; 95% CI [.38–.92]; *p* = .02). As the Group × Education interaction was significant (*p* = .03), interpretation focuses on the within‐education contrasts; relative to HG, LHG was associated with higher odds of high intention among lower‐educated patients (OR = 2.55, 95% CI [1.03, 6.27], *p* = .04) and medium‐educated patients (OR = 2.47, 95% CI [1.15, 5.30], *p* = .02), whereas there was no difference among high‐educated patients (OR = .98, 95% CI [.71, 1.34], *p* = .88) (Figure 2). The overall model fit was significant (*χ* ^2^ (15) = 41.68; *p* < .001).

Furthermore, meaning in life showed a significant main effect on intention‐to‐change‐lifestyle (OR = 2.03; 95% CI [1.48–2.76]; *p* < .001), indicating that patients with higher perceived meaning in life were more likely to exhibit a high intention‐to‐change‐lifestyle. Main effects of other factors were not significant.

Table 3 displays the OR and 95% CI from the logistic regression analysis predicting high intention‐to‐change‐lifestyle.

**TABLE 3:**
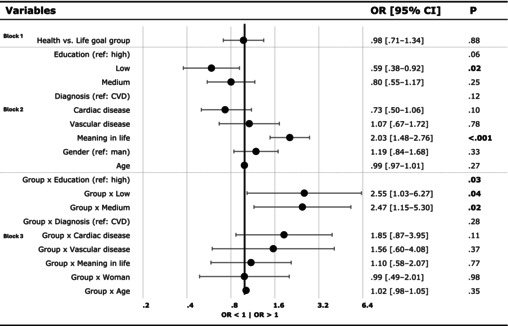
Odds ratio for high vs. low intention‐to‐change‐lifestyle.

*Note*: Group = goal‐setting condition (0 = health‐goal‐group, HG; 1 = life‐and‐health‐goal‐group, LHG). Education = education level. Diagnosis = cardiovascular diagnosis. CVD = cardiovascular disease. Logistic regression was entered in three blocks: Block 1 (goal‐setting condition: HG vs. LHG); Block 2 (main effects of socio‐demographic/health variables); Block 3 (interactions between condition and those variables). For clarity, only effects from the relevant block are shown together. The x‐axis shows direction: OR >1 indicates higher odds of high intention; OR <1 indicates lower odds.

## DISCUSSION

This study examined goal setting in CVD patients. In brief, three patterns emerged. Patients mostly preferred life goals focused on their personal health, their family and their relationship. Regarding health goals, exercise goals were most frequently selected, by over two‐thirds of patients. When life goals were linked to health goal setting, the prevalence of selecting stress management as a health goal was higher. Contrary to our primary hypothesis, we found no significant direct impact of goal‐setting condition on patients' intention‐to‐change‐lifestyle, and we therefore reject the primary hypothesis of a main effect. In exploratory analyses, the linkage between life and health goals was associated with more than twofold higher odds of high intention among lower‐ and medium‐educated patients.

The higher selection of stress management is clinically relevant as CVD patients frequently experience a broad spectrum of psychological distress, which has been shown to adversely impact their well‐being (Tawakol et al., 2017) and can act as a trigger for acute CVD events (Chinnaiyan, 2019). Therefore, addressing stress management as part of a comprehensive approach to managing CVD and promoting better health outcomes is highly advisable (Cohen et al., 2016; Schaefer et al., 2013). Our findings also underscore the nuanced nature of goal setting in CR, emphasizing the need to tailor strategies to encompass both medical and psychosocial aspects of patient care.

In contrast to our hypothesis, we observed no overall difference between HG and LHG in intention‐to‐change‐lifestyle. This may be due to high post‐task intention in our sample, as well as sample characteristics, such as higher well‐being, older age and higher education (Kobau et al., 2010); hence, adding an explicit life goal may have provided little additional benefit over standard health‐goal setting. Still, further exploratory analyses indicated that linking life and health goals may be most beneficial for patients with lower and medium education levels, as it was associated with higher intention‐to‐change‐lifestyle in these groups, but not among those with high education. This aligns with research suggesting that personalization and contextualization of health goals can motivate lifestyle changes (Antonoplis & Chen, 2021; Arnold et al., 2012; Kim et al., 2019; Lampert et al., 2014; Schaefer et al., 2013). An explanation may be that daily demands and stressors may compete with attention to abstract, higher‐order goals for some patients, particularly those with lower education levels (Antonoplis & Chen, 2021; Bukman, 2016). This, however, can lead to damaging health‐related decisions in the long term (Sheehy‐Skeffington & Rea, 2017). Consistent with this, patients with lower education were less inclined to demonstrate high intention than those with higher education. Making the link between life and health goals explicit might help translate personal values into concrete behavioural steps for this population. This tentative interpretation requires confirmation, including replication of subgroup findings, to determine whether effects extend beyond intention‐to‐change‐lifestyle.

Finally, several socio‐demographic or health‐related factors impacted patients' intention‐to‐change‐lifestyle. Higher perceived meaning in life showed a robust main effect, as it was associated with a twofold higher odds of high intention and did not interact with group, indicating a consistent association across both conditions. This highlights the importance of patients' having a sense of purpose when motivating them to pursue lifestyle changes. However, it is also possible that a lower sense of meaning in life is associated with increased depressive symptoms, which are highly prevalent among cardiovascular patients (Huffman et al., 2013). Given that depressive symptoms are known to reduce motivation for lifestyle changes, they may partially explain the observed association between meaning in life and intention to change lifestyle. Future studies should include depression screening to clarify this link. Age and gender neither predicted intention nor moderated the group effect.

Taken together, these findings suggest the potential value of explicitly linking life and health goals in routine consultations. In line with patient‐centred health care (Rossi et al., 2023), by structuring conversations to deliberately relate life goals to health goals, health care professionals can create a more personalized health care plan to align treatment with individual priorities to encourage patients to commit to lifestyle changes, thereby potentially improving the efficacy of the management of CVD. Such tailoring is particularly important in behaviour‐change programmes, where goal setting is a cornerstone technique (Epton et al., 2017), and differs by socioeconomic position (SEP) (Spring et al., 2021). In CR, the same pattern is seen: lower education, often a marker of lower SEP, relates to poorer perceived health and outcomes (Antonoplis & Chen, 2021). People with lower SEP report worse perceived health and health outcomes across multiple measures compared to those with a higher SEP (Skýbová et al., 2021). This discrepancy is concerning, as people with lower SEP are underrepresented, drop out more, and benefit less when they do participate (Bukman, 2016; Western et al., 2021). Our results add a new dimension to this research by revealing that patients with lower and medium education levels were associated with 57% and 44% lower odds, respectively, of having a high intention to change their lifestyle than those with higher education. This underscores the need for targeted, personalized goal‐setting approaches to effectively address and reduce socioeconomic health disparities (Coupe et al., 2018; Visseren et al., 2021).

### Strengths and limitations

Our study had several strengths and limitations. We examined a large sample of CVD patients across the Netherlands. The design pioneers the investigation of contextual factors that influence goal setting in real‐life cardiac care, helping to fill a significant knowledge gap. Finally, this study utilizes the current European Guidelines on Prevention of Cardiovascular Disease in Clinical Practice, with specific recommendations for goal setting in patients with CVD.

Concerning limitations, the sample was mostly men and highly educated, limiting the generalizability to other populations. While the gender distribution is representative of CR patients in the Netherlands (Vanhommerig et al., 2024), the educational background may not be. Second, the Wat ertoe doet tool, used in the LHG group, may have facilitated structured reflection, potentially influencing engagement. Third, health goals were categorized based on the observable behaviour described, regardless of the lifestyle domain selected by the participant. While this behaviour‐based coding allowed for consistent analysis of goal content across participants, it may obscure how participants themselves intended to link certain behaviours with specific lifestyle domains. Future research could analyse domain choice and behavioural goal content separately to better understand these subjective linkages. Fourth, the HG group functioned as an active usual‐care control; without a no‐goal or information‐only group, absolute effects versus no intervention cannot be estimated. In addition, we did not include a self‐report manipulation check; future studies should add a brief item to verify perceived linkage. We also measured Meaning in Life only post‐task, which precludes mediation tests and limits inferences about change; future studies should assess Meaning in Life both pre‐ and post‐task and include longitudinal behavioural outcomes. Fifth, intention‐to‐change‐lifestyle was already high at the single post‐task assessment in our sample. While intention is a known predictor of behaviour (Sheeran, 2002), there is an intention‐behaviour gap (Sheeran & Webb, 2016). Future research could assess whether linking life goals to health goals translates into measurable health outcomes (e.g., weight loss, blood pressure improvements or fitness levels) (Hettema et al., 2005). Finally, to limit burden we did not assess current behaviours, time since diagnosis or CR participation, and, as an online experiment, we lacked detailed clinical data (e.g., BMI, comorbidities, prior rehabilitation). Including these factors in future studies will help optimize timing and clarify effectiveness across patient subgroups.

### Future directions

Successful implementation of goal‐setting strategies in CR requires a structured life‐goal tool (e.g., *Wat ertoe doet*) that supports both patients and health care providers, with attention to intake procedures, staff training and optimal timing within the CR pathway. Future studies should include an information‐only/no‐goal arm to estimate the absolute added value of goal setting.

Our findings also align with Motivational Interviewing (MI), which links behaviour change to personal values and goals. Explicitly linking life and health goals may enhance intrinsic motivation, particularly among lower‐ and middle‐educated patients, consistent with their higher intention‐to‐change‐lifestyle. Similar to MI, this approach empowers patients to take an active role in managing their health, including factors such as stress levels. This, in turn, may increase the likelihood that intention translates into action. However, as seen in MI interventions, an intention‐behaviour gap remains. Future work should test life‐health goal linkage combined with goal monitoring, feedback, cognitive restructuring and stress management to optimize outcomes. As with MI preceding more intensive therapy, this goal‐setting method could serve as a first step in personalized CR before interventions such as Cognitive Behavioural Therapy (Miller, 2010), setting the stage for more profound behaviour change.

Finally, our findings share similarities with concepts from structured stress‐management programmes (Prochaska et al., 2012), as our approach focuses on improving well‐being by integrating personal life goals into behaviour change strategies. The greater uptake of stress‐management goals in the LHG suggests potential benefits for stress‐related outcomes. Future research could test our goal‐setting approach in combination with validated stress‐management techniques (e.g., mindfulness‐based stress reduction) to enhance CR effectiveness.

## CONCLUSION

While the direct effect of goal‐setting condition on intention‐to‐change‐lifestyle was non‐significant, this study provides valuable insights for cardiac clinical practice. It suggests the potential utility of personalized goal‐setting approaches in facilitating lifestyle changes in patients with CVD. We observed practice‐relevant signals: life‐goal linkage was associated with a higher prevalence of selecting stress management, and exploratory analyses showed a higher intention‐to‐change‐lifestyle among patients with lower and medium levels of education. This may imply a need for more structured conversations between health care professionals and patients, especially for those with lower and medium education levels. This individualized approach to health care may enhance disease management and overall patient well‐being. To implement personalized goal‐setting strategies within clinical practice, health care providers benefit from practical information on goal setting at different hierarchical levels, its interconnecting impact and the significance of personalized, value‐based goal setting for different patient groups.

## AUTHOR CONTRIBUTIONS

**Renée V. H. IJzerman:** Conceptualization; investigation; writing – original draft; methodology; visualization; writing – review and editing; software; formal analysis; project administration; data curation; validation. **Rosalie van der Vaart:** Conceptualization; supervision; data curation; formal analysis; writing – review and editing; writing – original draft; visualization; methodology; software; validation; resources. **Linda D. Breeman:** Writing – review and editing; visualization; methodology; formal analysis; data curation; supervision; writing – original draft; conceptualization; validation. **Inge van den Broek:** Writing – review and editing. **Elise Dusseldorp:** Methodology; writing – original draft; writing – review and editing; visualization; formal analysis; data curation; validation. **Roderik Kraaijenhagen:** Writing – review and editing; funding acquisition. **Thomas Reijnders:** Writing – review and editing; conceptualization. **Andrea W. M. Evers:** Conceptualization; methodology; supervision; data curation; formal analysis; visualization; validation; funding acquisition; writing – original draft; writing – review and editing; software; resources. **Wilma J. M. Scholte op Reimer:** Conceptualization; methodology; supervision; data curation; formal analysis; visualization; writing – review and editing; validation; writing – original draft; funding acquisition; software; resources. **Veronica R. Janssen:** Conceptualization; funding acquisition; writing – original draft; writing – review and editing; visualization; methodology; validation; formal analysis; data curation; supervision; software; resources.

## FUNDING INFORMATION

This work was supported by The Netherlands Cardiovascular Research Initiative: An Initiative with the support of the Dutch Heart Foundation, CVON2016‐12 BENEFIT, and ZonMw (The Netherlands Organization for Health Research and Development) and members of the BENEFIT consortium.

## CONFLICT OF INTEREST STATEMENT

The authors declared no potential conflicts of interest concerning this article's research, authorship or publication.

## Supporting information


Data S1:

## Data Availability

The data underlying this article will be shared on reasonable request to the corresponding author.
