# Supplementary material for: What matters to cardiac patients? The impact of linking life goals to health goals on patients' intention‐to‐change‐lifestyle: an online experiment
Source: Br J Health Psychol. 2026 Jan 30;31(1):e70056. doi: 10.1111/bjhp.70056 (PMC12857248; doi:10.1111/bjhp.70056)
Supplement: Supplementary file 1 — Data S1: [file BJHP-31-0-s001.docx]

**Appendix S1.**

***What matters* tool by Harteraad (2013)**

**What matters**

"NOW I **know** **what matters** to me!"

Answer the **4 questions** on this website. Then, you will know better **what matters to you** in your life.

This helps when you **talk** to your **healthcare provider** and the people around you. This way, you can make **choices** more easily. And receive the **healthcare** that **suits your life NOW**.

Start the questions!

**Question 1**

What is important to me in life?

**Question 2**

What is important to me in life NOW?

**Question 3**

What do I want to be able to do?

**Question 4**

What do I need to be able to do this?

*After clicking on the ‘Start the questions' button, the first question appears on a new screen.*

**Question 1. What is important to me in life?**

**View** the **9 topics**. **Click** on up to **3 images**.

**My family My personal health**

**
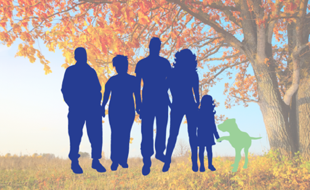

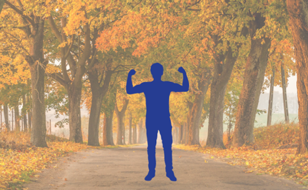
**

**My well-being My home and neighbourhood**

**
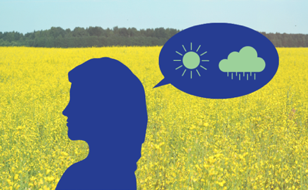

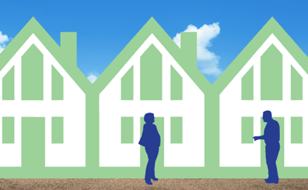
**

**My daily life My independence**

**
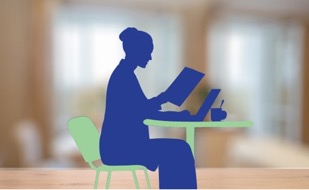
**
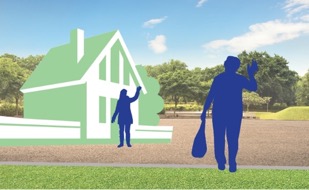


**My relationships My friends**

**
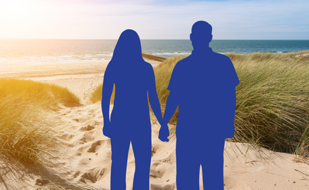

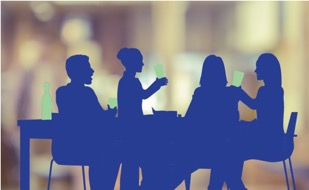
**

**My personal growth**

*
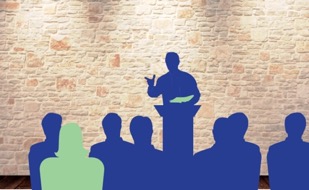
*

*After selecting up to three images,
for instance, ‘My personal health’
and ‘My independence, and clicking
the button, it second question
appears in a new screen.*

**Question 2. What is important to me in life NOW?**

**Click** on **1 image**.*

**My personal health My independence**

**
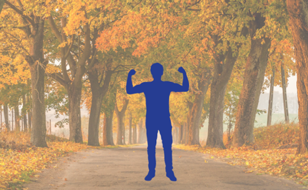
**
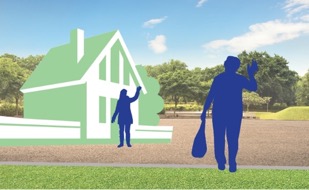


**All selected options from Question 1 are displayed, for example, ‘My personal health’ and
‘My independence’.*

*After selecting one final option, for example, ‘My personal health’, and clicking the
‘Next question' button, the third question appears on a new screen.*

**Question 3. What do I want to be able to do?**

**'My personal health'** is important to me in life **NOW**.

Type below what you want to be able to do:

Examples from others*

“Picking up my sport.”

“Being able to walk the dog.”

“Cycling to school with my child.”

**When typing something in the yellow text box, all given examples disappear.*

*After typing an answer in the yellow bar, for example, ‘Walking in the woods with my family.’
and clicking on the ‘Next question’ button, the last question appears on a new screen.*

**Question 4. What do I need to be able to do this?**

**Click** on up to **2 images**.

**Having energy Being able to exercise**

**or play sports**

**
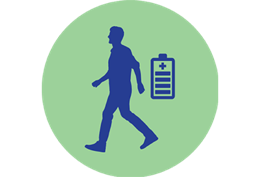

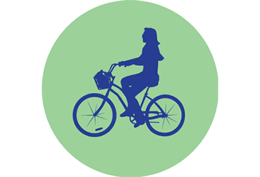
**

**Feeling relaxed Being content with myself**

**
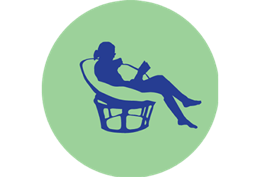

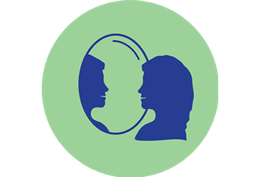
**

**Speaking fluently Thinking clearly and being**

**able to remember things**

**
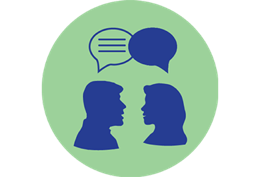

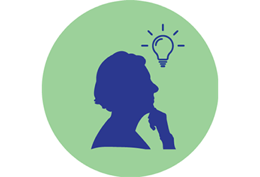
**

**Feeling confident Being able to do things
 by myself**

**
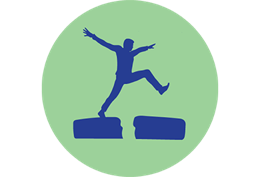

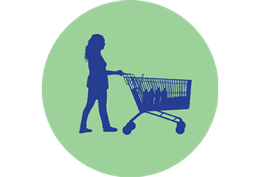
**

**Having fewer symptoms Being able to have fun**

**
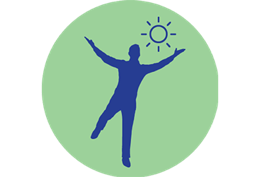
**

**
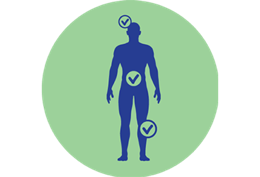
**

*After selecting 1 or 2 images and clicking on the ‘Next question’-button, the final page of the tool appears.*

**Overview: What matters in my life**

You're done! Below are the **answers** to questions 1 to 4.

You can also **type** in **comments**.

**Keep** the summary for **yourself**.

Or **talk** about it with your **healthcare** **provider** and the **people around you**.


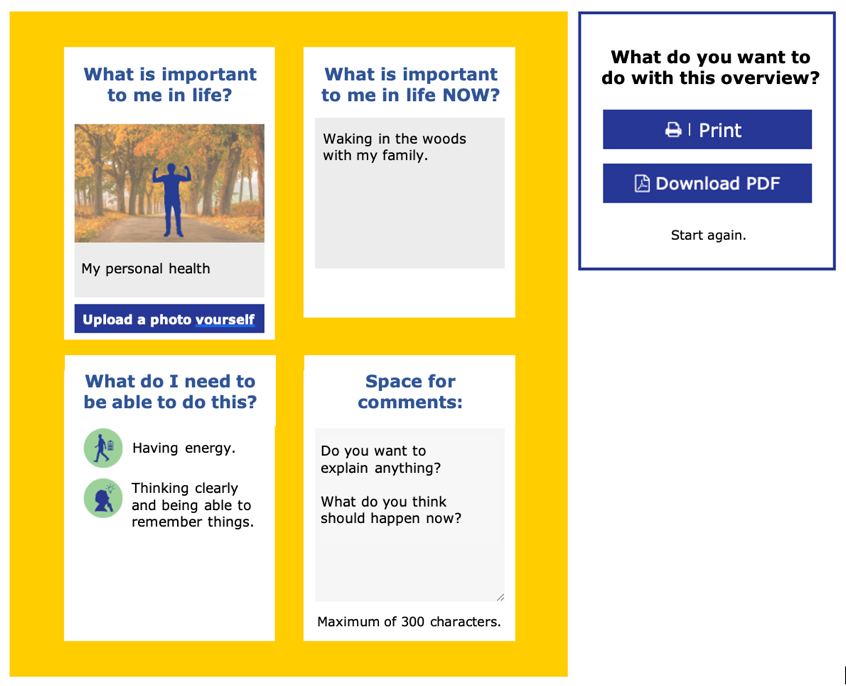


✅ Discuss ‘What Matters’ with your loved ones
✅ Keep the printout in a visible place (message board, fridge door, home screen laptop...)

✅ Every now and then reflect on whether you are doing enough of what matters to you
✅ Repeat the 4 steps if something else is (also) important in your life now
✅ Take the printout to your healthcare professional
✅ Discuss it together, explain where necessary
✅ Keep the printout as a basis for further discussion

**Appendix S2.**

**Skewed distribution of the main outcome intention-to-change-lifestyle.**

**SPSS output:**

| **Intention-to-change-lifestyle** | | | | | |
| --- | --- | --- | --- | --- | --- |
|  | | Frequency | Percent | Valid Percent | Cumulative Percent |
| Valid | .00 | 3 | .5 | .5 | .5 |
|  | 1.00 | 1 | .2 | .2 | .6 |
|  | 2.00 | 1 | .2 | .2 | .8 |
|  | 2.50 | 1 | .2 | .2 | 1.0 |
|  | 3.00 | 1 | .2 | .2 | 1.1 |
|  | 3.50 | 3 | .5 | .5 | 1.6 |
|  | 4.00 | 1 | .2 | .2 | 1.7 |
|  | 4.50 | 1 | .2 | .2 | 1.9 |
|  | 5.00 | 14 | 2.2 | 2.2 | 4.1 |
|  | 5.50 | 10 | 1.6 | 1.6 | 5.7 |
|  | 6.00 | 17 | 2.7 | 2.7 | 8.4 |
|  | 6.50 | 10 | 1.6 | 1.6 | 10.0 |
|  | 7.00 | 56 | 8.9 | 8.9 | 18.9 |
|  | 7.50 | 40 | 6.4 | 6.4 | 25.3 |
|  | 8.00 | 134 | 21.3 | 21.3 | 46.6 |
|  | 8.50 | 40 | 6.4 | 6.4 | 52.9 |
|  | 9.00 | 91 | 14.5 | 14.5 | 67.4 |
|  | 9.50 | 16 | 2.5 | 2.5 | 70.0 |
|  | 10.00 | 189 | 30.0 | 30.0 | 100.0 |
|  | Total | 629 | 100.0 | 100.0 |  |

**
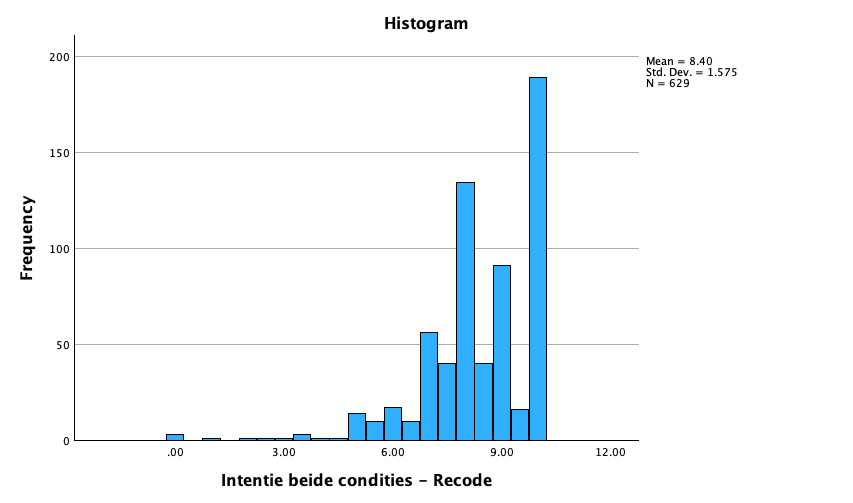
**

**Appendix S3.**

**Chosen life goal domains and life goal examples in the life-and-health-goal-group (n = 306).**

| **Life goal domain** | ***n* (%)** | **Examples of formulated life goal** |
| --- | --- | --- |
| *My personal health* | 106 (34.6) | - “Being able to move well.” - “Being able to continue doing what I have been doing.” - “Living without physical discomfort.” - “Being able to fully resume my work and still have energy to spare.” - “I want to improve my physical condition to make it easier to recover   after physical activity.” |
| *My family* | 97 (31.7) | - “Being and remaining an active mother and grandmother.” - “Seeing my grandchildren grow up.” - “Being there for my family members in times when things are not going   well, enjoying when things are going well, enjoying my grandchildren.” - “Creating a beautiful life with my family.” - “Keeping in touch with my kids and grandkids often.” |
| *My relationship* | 36 (11.8) | - “Paying enough attention to each other.” - “Growing old together.” - “Taking care of my partner when they need it and allowing   her to care for me when necessary. Looking out for each other   and enjoying our time together.” |
| *My well-being* | 29 (9.5) | - “I want to feel relaxed and free to fill my days. No stress, no harm.” - “Having more mental energy, feeling relaxed and happy. From there,   other things that I find important come into motion. Otherwise, it will   not work in those areas either.” - “Feeling calm and balanced.” |
| *My independence* | 17 (5.6) | - “Being able to go anywhere and meet people, meet again for outside   drinks, and hold meetings not via the computer.” (Note: due to covid) - “Being able to go anywhere independently.” |
| *My daily life* | 13 (4.2) | - “Being meaningfully engaged in voluntary work.” - “Being in harmony with my surroundings.” |
| *My personal growth* | 5 (1.6) | - “Being able to follow a new training course.” - “Gaining more confidence in my faith.” |
| *My friends* | 2 (0.7) | - “Being in contact with friends frequently.” - “Being able to sing with my quintets. Was important, is   important, but I’m not able to right now.” (Note: due to covid) |
| *My home and neighbourhood* | 1 (0.3) | - “Staying at house and socialising with my neighbours during   this covid time.” |

**Appendix S4.**

**Examples of formulated health goals in both goal-setting conditions (N = 629).**

| **Health goal domain** | ***n* (%)** | **Examples of formulation of health goals** |
| --- | --- | --- |
| *Exercise* | 418 (66.5) | - “Doing something in the garden every day. Large garden!” - “One hour of daily exercise (walking, cycling, exercises).” - “Go for a walk every day. Doing morning exercises. Practising tai-chi.” - “Keeping my physical condition in good shape by walking, cycling   and eating healthy.” - "Exercising every day, walking in winter and cycling in summer.   Staying active and avoid sitting on the couch, running errands on foot   when I can.” |
| *Stress management* | 83 (13.2) | - “No phone or anything, just doing my thing without being bothered.” - “Enjoying nature, art and music.” - “Learning to let go of my work more by doing relaxation exercises or   joining a yoga group.” - “Building in daily moments of relaxation.” - “Taking more rest, not always going on and on.” |
| *Nutrition* | 91 (14.5) | - “Putting fewer tablespoons of food on my plate a few meals a week.” - “No eating fatty sweets, except on special occasions. Not indulging in too   many biscuits, chocolate, etc.” - “I do not eat meat, and almost everything is organic, few dairy products,   only cheese, hardly any alcohol, I do eat liquorice, but that is not good   for your blood pressure... well…” - “Eating fruit and vegetables every day, and don't overeat.” - “No more unhealthy snacks in between meals.” |
| *Sleep* | 15 (2.4) | - “Going to bed on time.” - "Do not start worrying when I wake up at night.” - “Sleeping through the night.” |
| *Alcohol cessation* | 12 (1.9) | - “No alcohol during the week.” - “Only an occasional glass of wine.” - “During the week, no more than one glass of alcohol a day.” |
| *Smoking cessation* | 10 (1.6) | - “Trying to smoke one cigarette less every day.” - “Quitting smoking.” - “Keeping up the habit of never lighting a cigarette again.” |

**Appendix S5.**

**Selected life goals and health goals divided by goal-setting condition.**

**
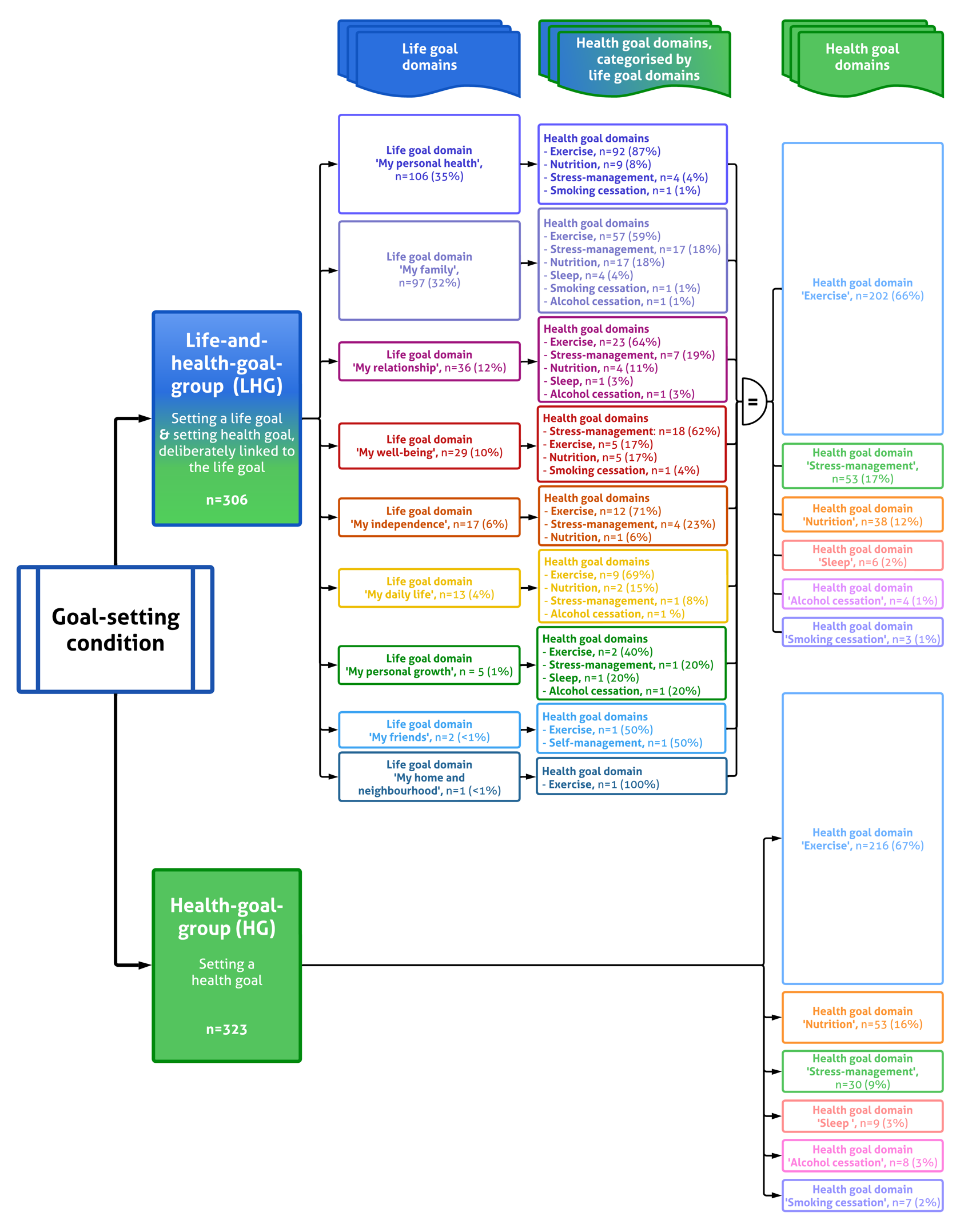
**
